# Case Report: Increased FGF23 and new insufficiency fractures at burosumab discontinuation in X-linked hypophosphatemia

**DOI:** 10.3389/fendo.2025.1665649

**Published:** 2025-10-23

**Authors:** Robert Bandir, Laura Zanisi, Marie Nicod-Lalonde, Elena Gonzalez-Rodriguez

**Affiliations:** ^1^ Internal Medicine Department, Ente Ospedaliero Cantonale, Locarno, Switzerland; ^2^ Faculty of Biomedical Sciences, Università della Svizzera Italiana, Lugano, Switzerland; ^3^ Department of Nuclear Medicine and Molecular Imaging, University Hospital of Lausanne and Lausanne University, Lausanne, Switzerland; ^4^ Interdisciplinary Center for Bone Diseases, Rheumatology Service, University Hospital of Lausanne and Lausanne University, Lausanne, Switzerland

**Keywords:** X-linked hypophosphatemic rickets, FGF23, burosumab, fracture healing, case report

## Abstract

Burosumab, a monoclonal antibody that binds and inhibits FGF23 activity, was approved in 2018 for treatment of children and adults with X-linked hypophosphatemia (XLH), an X-linked dominant disorder due to increased serum fibroblast growth factor 23 (FGF23) concentration. XLH presents with chronic hypophosphatemia that provokes rickets and dental complications in children, which persist in adults accompanied by bone pain, insufficiency fractures, and enthesis calcifications. Historically, treatment relied on oral phosphate and active vitamin D supplementation. Randomized clinical trials have shown that burosumab allows for hypophosphatemia correction and significant improvement of clinical symptoms in both children and adults. Moreover, fracture healing is 16.8 times higher compared with placebo-treated patients. However, optimal treatment duration has not been determined, and there are few data on clinical, biological, or radiological consequences of burosumab discontinuation. We present the case of a 36-year-old young woman with XLH and disability in the context of chronic bilateral femoral shaft fractures progressing despite optimal phosphate and calcitriol supplementation. After only three burosumab doses, fracture lines were no longer visible on X-rays and the patient could stop pain killers. Burosumab was interrupted after 11 months due to lack of insurance reimbursement. Three months after the last injection, a bone scintigraphy performed because of thigh pain recurrence showed healing of previous bilateral femoral fractures and showed the development of a new fracture on the right femoral shaft, in the presence of very high intact FGF23 values (9,330.0 pg/ml; N 10-50; patient values without treatment: 91.4 pg/ml). While burosumab may interfere with FGF23 dosage during treatment, it should be nearly totally eliminated after ≤95 days (half-life ≤19 days), suggesting that FGF23 accumulated under burosumab inducing a very rapid relapse of clinical symptoms. Because in some cases burosumab treatment should be interrupted (end of reimbursement, pregnancy in the absence of safety data), further studies are needed to better explain the FGF23 increased levels after burosumab discontinuation and the clinical, biological, and radiological consequences of burosumab withdrawal.

## 1 Introduction

X-linked hypophosphatemia (XLH) (OMIM 307800; ORPHA 89936) accounts for approximately 80% of inherited hypophosphatemic rickets, with an estimated prevalence of 1.7-4.8/100,000 (1, 2). XLH due to loss-of-function pathogenic variants in the phosphate-regulating gene with homology to endopeptidase (PHEX) located on Xp22.1 is inherited with an X-linked dominant pattern. Pathogenic variants in the *PHEX* gene lead to excess of intact fibroblast growth factor 23 (iFGF23), which induces chronic hypophosphatemia via decreased renal tubular phosphate reabsorption and decreased gastrointestinal phosphate absorption secondary to decreased 1,25-dihydroxyvitamine D [1,25(OH)_2_D] production (1). Patients with XLH usually present with rickets and odontomalacia in children, which persist in adults who further develop osteomalacia, with a broad phenotypic spectrum. Main clinical symptoms are growth retardation and short stature, gait disturbances due to lower limb deformities, and joint stiffness and bone pain (reported in 45% of adult patients). Moreover, adult patients with XLH are at risk of complications such as early osteoarthritis, enthesopathies, spinal stenosis, fractures or pseudofractures (up to 52%), hearing loss and limiting quality of life (1, 2).

Conventional treatment with oral supplementation of phosphate and active vitamin D analogs (1, 2) has limited clinical impact, its main effects being rickets healing, limiting dental abscess formation, and preventing progressive growth failure. However, frequent dosing and tolerability issues (gastrointestinal discomfort) may decrease adherence, and in a substantial proportion of patients, it is unsuccessful and/or associated with adverse effects including hypercalciuria, nephrocalcinosis, and secondary or tertiary hyperparathyroidism (3, 4). Thus, the benefit/risk profile for long‐term conventional therapy in adults with XLH is uncertain, and treatment in adults is not systematic. Current recommendations (2) advise to treat only symptomatic patients for bone pain, insufficiency fractures or pseudofractures, chemical evidence of osteomalacia (i.e., elevated alkaline phosphatase rates), planned orthopedic procedures, or pregnancy and lactation. Since 2018, the Food and Drug Administration (FDA) and the European Medicines Agency (EMA) approved treatment of XLH with burosumab, a fully human monoclonal IgG antibody that binds and inhibits FGF23. Consistent evidence in multiple trials in children and adults shows that burosumab treatment is associated with improvement in phosphate homeostasis and healing of rickets and osteomalacia (5). More specifically, in adults, burosumab reduced the Western Ontario and the McMaster Universities Osteoarthritis Index (WOMAC) stiffness and physical function subscales, as well as Brief Pain Inventory by MD Anderson Center (BPI) worst pain, and increased the odds of healed fracture by 16.8‐fold compared with placebo (p < 0.001) in only 24 weeks (4), with a similar safety profile. However, there is no consistent data to assure long-term efficacy and safety of burosumab or determine the optimal treatment duration or the consequences of burosumab therapy discontinuation.

We describe the case of a young patient for whom burosumab treatment had cured femoral shaft chronic long-standing osteomalacic fractures and disabling symptoms that had progressed despite conventional treatment. Burosumab discontinuation provoked severe relapsing of clinical, biological, and radiological consequences of XLH.

## 2 Case description

**Figure 1:**
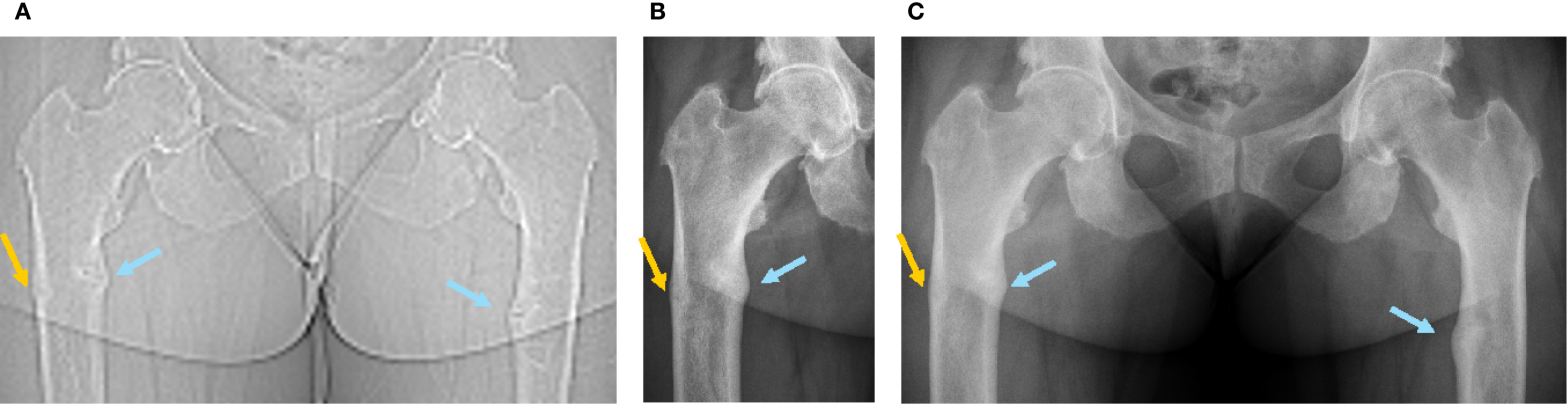
Radiographic images of the femoral fractures before and after introduction of burosumab treatment (first burosumab injection: 04.01.2021). **(A)** SPECT-CT topogram before introduction of burosumab treatment (16.09.2020). **(B)** Five weeks after first burosumab injection (12.02.2021). **(C)** Three and a half months after first burosumab injection (23.04.2021).

**Figure 2:**
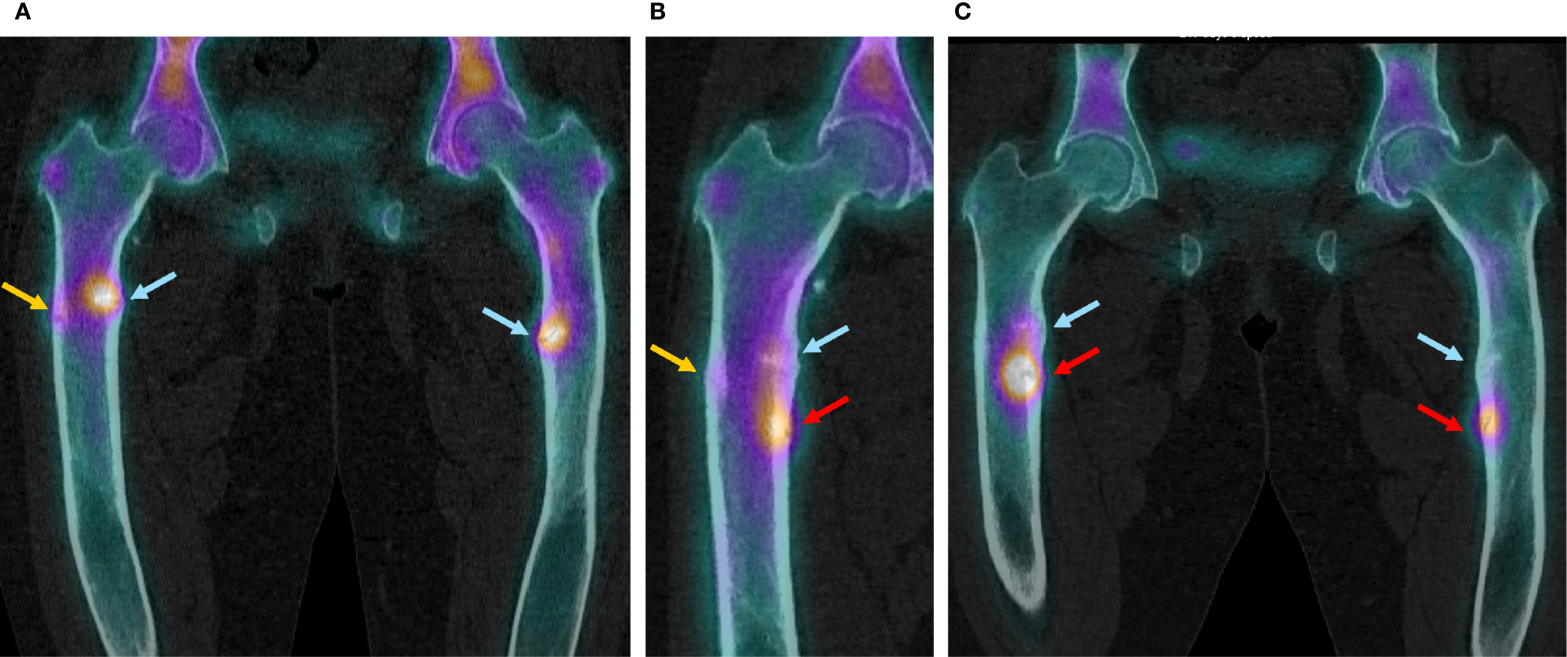
SPECT-CT images of the femoral fractures before introduction of burosumab treatment and after its discontinuation. **(A)** Before introduction of burosumab treatment (16.09.2020). **(B)** Three months after last burosumab injection (10.01.2022). **(C)** Eleven months after last burosumab injection (14.09.2022).

A 36-year-old woman was followed at our consultation for XLH due to a *de novo* heterozygote variant on the exon 16 of the PHEX gene. First symptoms that appeared during childhood required orthopedic surgeries of both tibia for bowing legs despite early introduction of conventional treatment; she developed *coxa vara* and *profunda* bilaterally. After a period of bad adherence to phosphate substitution due to digestive secondary effects, she developed femoral shaft bilateral pseudofractures of the medial cortical (incomplete cortical fracture on radiography associated with periosteal reaction) at ages 24 (left) and 26 (right) years (**Figures 1A**, **2A**, blue arrows), which were managed conservatively with good initial response thanks to improved treatment observance after a phosphate formula change, and weight discharge. They however did not completely heal, with occasional transient increase in pain, and progressive appearance of joint stiffness, depending on intermittent irregular observance.

**Figure 3:**
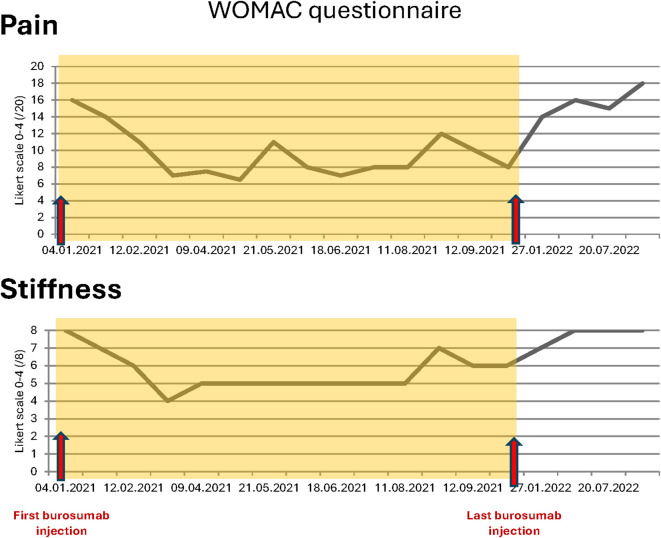
WOMAC questionnaires total scores for pain and stiffness during and after burosumab treatment.

In September 2020, she went on sick leave because of increasing leg pain that affected sleeping time and increasing disability in her daily life activities despite optimal calcitriol and phosphate supplementation, with minimal doses because of digestive intolerance. She used two rods to ambulate, and standard pain killer medications did not relieve symptoms as assessed by the WOMAC questionnaire in January 2021 before burosumab treatment (**Figure 3**).

Blood exams showed slightly decreased phosphate serum concentrations (0.73 mmol/L; N 0.80-1.40), with alkaline phosphatase and 25-OH vitamin D in the normal range and mild secondary hyperparathyroidism (**Table 1**, first row). Renal function showed a normal result (creatinine 50 µmol/l, N 44-80). As expected, iFGF23 was elevated (**Table 1**), and there was renal phosphate wasting. SPECT-CT showed a new threatening fracture of the lateral cortical of the right femur (**Figures 1A**, **2A**, orange arrow) and highly increased activity on the two already known ones. Since bowing legs favor insufficiency fractures, orthopedist advise was to realign the femurs to decrease constraints and risk of spontaneous completion. At this point, a request for reimbursement of burosumab, which was available in Switzerland since May 2020, was transitorily accepted by her health insurance. The patient received the first dose on 04.01.2021, after 2 weeks of conventional treatment washout. It was administered every 4 weeks with incremental doses in order to approach the lower limit of normal (LLN) phosphate serum concentration 2 weeks after an injection.

**Table 1:** Biological results of phospho-calcic metabolism parameters before, during, and after burosumab treatment.

Date	Treatment	Cac (mmol/L) N: 2.10-2.50	P (mmol/L) N: 0.80-1.40	ALP (U/L) N: 36-108	TmP/GFR (mmol/L) N: 0.80-1.35	Intact FGF23 (pg/mL) N: 10-50	25-OH vit D (µg/L) optimal: 20-30	1,25 (OH)_2_ vit D (pmol/L) N:48-190	Intact PTH (ng/l) N:10-70
**31.08.2020**	Phosphate + calcitriol	2.08	0.72	96	–	135.1	31.0	–	72
**16.09.2020**	Diagnosis of a new threatening fracture of the lateral cortical of the right femur (**Figures 1A**, **2A**)								
**27.10.2020**	Phosphate + calcitriol	2.16	0.81	–	0.31	–	22.4	–	–
**04.01.2021**	2 weeks wash out	2.15	0.40	133	0.31	91.4	16.0	65	70
**15.01.2021**	Burosumab 60 mg 04.01	2.22	0.72	107	0.63	>800	16.5	298	69
**12.02.2021** **23.04.2021**	Healing of chronic femoral shaft bilateral pseudofractures (**Figures 1B, C**)								
**27.08.2021**	Burosumab 90 mg 11.08	2.15	0.66	104	–	>84’000	–	264	–
**13.09.2021**	Burosumab 90 mg 11.08	2.20	0.54	115	0.39	–	30.8	–	54
**10.01.2022**	No treatment*	2.18	0.33	94	0.26	9330	22.4	53	–
**10.01.2022**	New fracture on the right medial diaphyseal femur (**Figure 2B**)								
**28.02.2022**	Phosphate + calcitriol	2.14	0.40	99	0.43	826.7	–	109	–
**14.06.2022**	Phosphate + calcitriol	2.31	0.67	–	–	–	32.4	–	–
**14.09.2022**	New fracture on the left medial diaphyseal femur (**Figure 2C**)								

*Last burosumab injection on October 11th, 2021; phosphate and calcitriol reintroduced at this time-point.

Cac, Albumin-corrected calcium; P, phosphate; ALP, alkaline phosphatase; TmP/GFR, ratio of tubular maximal reabsorption of phosphate (TmP) to glomerular filtration rate (GFR); FGF23, fibroblast growth factor 23; 25-OH vit D, 25-hydroxy vitamin D; 1,25(OH)2 vit D, 1,25 dihydroxy vitamin D; PTH, parathormone. -: not assessed.

Under burosumab treatment, values of most biologic markers were comparable with those under conventional treatment, except for the increase in 1,25(OH) vitamin D (**Table 1**); iFGF23 also increased over the range capacity of the laboratory test, which was considered a preanalytic interference with burosumab treatment as suggested by the literature (see discussion). Despite maximal burosumab dosage (90 mg/month since mid-august 2021), neither phosphate levels nor phosphate resorption ever attained normal values 2 weeks after the injection and were significantly altered at the nadir just before next injection.

The most remarkable effects were related to the improvement of the patient’s symptoms. The function and pain questionnaires evaluated respectively by the WOMAC scores showed a rapid improvement in pain, stiffness, and disability (**Figure 3**). The patient stopped regularly taking painkillers 2 months after the beginning of burosumab, and she was able to resume part-time work. These findings were strictly related to radiological improvement: the fracture traits healed after two doses of burosumab (**Figures 1B, C**). On October 2021, the orthopedics renounced to the surgery because of closure of fracture trait and in part realignment of the femur.

Following clinical and radiological improvement, burosumab reimbursement was interrupted by the health insurance; last injection took place on October 11, 2021. Six weeks after last injection, the patient described pain and stiffness recurrence needing pain killers. On January 2022, 12 weeks after last injection, serum phosphate dropped to the lowest measured levels (**Table 1**) and iFGF23 measured levels were 100 times over usual patient values; conventional therapy with phosphate and calcitriol was restored at this point. iFGF23 stayed higher than usual for at least 6 more weeks (**Table 1**). A SPECT-CT was performed (**Figure 2B**) and showed the near total healing of the previous bilateral femoral fractures, but a new possible medial fracture on the right femur distally to the precedent one (red arrow). Despite maximally tolerated phosphate and calcitriol dosages, a new SPECT-CT in September 2022 (**Figure 2C**) showed a radiological worsening of this new fracture with a new additional fracture on the left medial diaphyseal femur (red arrows). Clinical evolution regressed with WOMAC scores comparable with those before the introduction of burosumab treatment (**Figure 3**). Of note, except for dental issues, the remaining renal, cardiac, auditory, and spinal investigations did not show common complications of XLH during the whole follow-up.

In November 2022, a compassionate treatment from the pharmaceutic agency was obtained a compassionate treatment from the pharmaceutic agency. Two weeks after the first burosumab injection on 21.11.2022, the serum phosphate concentration reached the LLN. Leg pain improved enabling pain-killer medication tapering, fewer nocturnal awakenings, and stiffness amelioration. New radiologic investigations on January and March 2023 showed a near complete healing of the two new fractures observed in the last SPECT-CT (09.2022).

## 3 Patient perspective

I received my first burosumab injection in December 2020. At that time, I was suffering from widespread pain in my joints, and many everyday gestures were impossible for me to perform. The pain was literally exhausting me on a daily basis, taking away my very will to live. After a few injections, the treatment radically changed my life as a woman, wife, mother, and professional. Then, in autumn 2021, I stopped treatment. Only a few weeks later, all hell broke loose. The pain returned very quickly and with greater intensity than before. It suddenly disabled me severely and was simply excruciating day and night. New fractures appeared. I was back on crutches … Afraid to mix with people for fear of being pushed around and suffering even more pain. I stood most of the time, because I needed a lot of time to get up and move on. No one could come near me or touch me, not my husband, not my daughter, not anyone. I needed daily help with the most common tasks, despite the painkillers (mefenamic acid, CBD and tramadol). When I benefited from the treatment again in November 2022, I was in a much worse state than before the very first injection. After 2–3 months, at the beginning of 2023, the pain had clearly diminished again, and the new fractures were almost completely healed. Today, I can say that I have a “normal” life, despite some pain which is mostly manageable without painkillers, and despite stiffness which limits me in certain daily gestures. For me, this treatment is not a way of improving my quality of life, but simply of living. I suffered the symptoms associated with the abrupt discontinuation of burosumab and I hope never to have to go through that again.

## 4 Discussion

We present the case of an adult patient with XLH who developed a new femoral shaft pseudofracture only 2 months after stopping burosumab given for the previous 11 months. She developed a second one 9 months later despite phosphate and calcitriol substitution. Thanks to burosumab, the patient had rapidly healed previous chronic long-standing diaphyseal shaft incomplete femoral fractures. Intact FGF23 measure, which was high during burosumab treatment, stayed >10 times above the patient’s usual values more than 3 months after last injection, accompanied by a rapid return to low phosphate and 1,25 OH vitamin D levels and clinical deterioration.

Patients’ evolution after burosumab discontinuation has been scarcely described. Two studies in adult patients reported on individuals who experienced a treatment gap between the main trial and the open-label extension phase (6, 7). In both of these studies, biological parameters returned to pre-burosumab values in patients who discontinued it; exact timing is not described. Clinical outcomes were only specified for the seven patients with a treatment gap of 8 to 15 months between phase III trial and the respective extension study (6). WOMAC questionnaire scores returned to the levels before burosumab treatment. Two other studies on adolescent patients showed identical evolution (8, 9), except for 2 of the 12 patients that did not present clinical deterioration at burosumab discontinuation (9). None of the studies describe new pseudofractures. This clinical deterioration at burosumab discontinuation is relevant as, in most countries, burosumab is reimbursed mainly in children, and only until the end of skeletal growth or up to the 17th or 18th year of age. Reimbursement in adults is mostly limited to patients who sustained pseudofractures; the duration of treatment is not precise, but it is expected that reimbursement would be discontinued after healing as in the case of our patient. Another situation in which burosumab has to be interrupted in the absence of safety data is pregnancy. This means that most patients will have to stop burosumab at a given time, and clinical consequences of treatment interruption need to be known. Our patient presents with severe XLH and previous pseudofractures in the context of bowed legs, which have probably participated to the development of new pseudofractures at burosumab discontinuation. Although development of new pseudofractures in this context may not be frequent, there may be disastrous clinical consequences and at-risk patients must be described in order to get determine who needs to get burosumab indefinitely.

Lack of immediate introduction of phosphate and calcitriol immediately after burosumab discontinuation, but only once low phosphate was confirmed, could have played a role in the poor clinical evolution. It is well defined that, to avoid hyperphosphatemia, a washout of phosphate and calcitriol for 2 weeks is needed before introducing burosumab. However, the protocol to interrupt burosumab has not been studied. Precocious introduction of phosphate and calcitriol before the date of virtual next burosumab injection, or progressive weaning of burosumab, could be alternatives to decrease the risk of symptoms relapsing.

One difficulty to choose the moment to reintroduce phosphate and calcitriol is the fact that the delay to decreased phosphate (duration of burosumab efficacy) is not well described. Burosumab pharmacokinetics (PK) is similar to other IgG1 monoclonal antibodies, with a first-order absorption and a linear elimination. Burosumab half-life is considered between 13 and 19 days depending on the dosage, with linear clearance up to doses of 1 mg/kg (10). We can estimate a near total elimination of burosumab serum concentration around 80–95 days (i.e., five times the half-life) after the last administration. Indeed, phosphate was already very low in our patient 90 days after last injection, but at that point, she had already developed a new femoral shaft fracture, which means that burosumab efficacy was probably lost since several weeks. Also, at this moment, iFGF23 values were much higher that pre-burosumab measurement in our patient.

Circulating biologically active iFGF‐23 is cleaved to generate two biologically inactive N‐terminal and C-terminal fragments. FGF23 immunoassays can either detect the intact (full-length) FGF23 (iFGF23) or both the intact protein and C-terminal fragments (cFGF23). Assays to measure iFGF23 recognize epitopes within both the N-terminal and C-terminal domains, whereas those measuring cFGF23 only bind to epitopes within the C-terminal portion; available assays have been summarized by Trombetti et al. (2). Most are only available for research purposes. Both iFGF23 and the C‐terminal fragment exhibit rapid renal clearance and similar half‐lives (<1 h).

Because burosumab targets the N-terminal region of FGF23, it is expected that it interferes with iFGF but not with cFGF23 dosages. Two studies analyzing iFGF23 and cFGF23 assay performance in burosumab-treated patients confirmed this hypothesis (11, 12). In the Piketty et al. study, iFGF23 was measured using the Liaison (Diasorin) assay, whereas Ashrafzadez-Kian et al. used the MedFrontier assay (Minaris Medical Co). A C-FGF23 assay (Biomedica ELISA) and an assay from Immunotopics (Quidel Corporation) were used for cFGF23 measures. In an *in vitro* experiment in samples from patients with normal or high iFGF23 and no treatment, Ashrafzadez et al. showed a dose-dependent decrease of the measured iFGF23 concentrations when incubating them with increasing burosumab levels, whereas cFGF23 was not affected by burosumab (11). In both studies, cFGF23 values in patients’ samples were higher after burosumab treatment than before treatment, and Piketty et al. showed that there was no analytic interference for this test with corresponding dilution values. iFGF23 values were higher after burosumab as compared with before burosumab using the MedFrontier assay and lower with the Liaison assay (11, 12). Indeed, analytical interference without reliable values with progressive dilutions were shown with this last one in the presence of burosumab. In conclusion, more reliable cFGF23 assays show strongly increased FGF23 levels in burosumab-treated patients, as does the MedFrontier iFGF23 assay despite the lowering effect of burosumab *in vitro*. The Kainos iFGF23 assay used in our clinic has not been evaluated for analytic interference but gives extremely high iFGF23 values too.

The reason for this high iFGF23 and cFGF23 levels in burosumab-treated patients is not known. The half-life of burosumab is similar after single subcutaneous administration of different dosages, and after multiple-dose administration, indicating that its elimination is not dose dependent (13, 14). Also, no difference was found between children and adults, without any factor modifying it, including baseline FGF23 levels (15). The very short half-life of FGF23 (<20 min (16–18)) makes that it should not affect its levels except if burosumab inhibits metabolization. While laboratory interference when on burosumab could justify very high values during treatment, it does not explain why they were still high after burosumab complete clearance (after 3 months, >5 times burosumab half-live, in the presence of very low phosphate and 1,25OH vitamin D).

Some hypotheses could be proposed. First, the reversibility of burosumab-FGF23 binding and the clearance of burosumab-bound FGF23 have not been studied as far as we know. One can imagine that iFGF23 has a lower elimination rate and a longer half-life in a burosumab-bound form, and if binding is reversible, it is progressively released with decreasing concentrations of burosumab when treatment is interrupted. To note, in two different long-time studies, it was found that there was a decreased efficacity of burosumab over time with increasing EC_50,t_ (serum concentration to reach 50% of maximum effect) (14, 19), whereas stable pharmacokinetics were described in another study (15). Zhang et al. suggested that it could be due to increases in total and unbound iFGF23 concentrations over pretreatment levels after burosumab administration. Actual very high measures with Kainos and MedFrontier iFGF23, and the cFGF23 assays, could represent this total (bound and unbound) measure.

Second, the elevation of iFGF23 and cFGF23 under burosumab treatment might reflect a maximized FGF23 production by osteocytes and osteoblasts with higher serum phosphorus levels (even if between normal range values), as proposed by Ashrafzadeh-Kian et al. (11).

In any case, accumulation of iFGF23 could explain the high measured levels after burosumab clearance, and the rapid recurrence of symptoms and of new fractures, rising the concern about the risk of burosumab treatment interruption specially in severely XLH-affected patients. Based on this thoroughly described single case, it is not clear whether we are dealing with a sort of rebound effect of burosumab treatment discontinuation or with a natural evolution of XLH disease after burosumab interruption.

Further studies are needed to better explain the FGF23 elevation after burosumab therapy and if this parameter could be used in clinical practice as a surrogate to establish the risk of pseudofractures after treatment discontinuation.

## Author’s note

X-linked hypophosphatemic rickets is a genetic disease characterized by chronic hypophosphatemia due to FGF23 elevated levels, usually treated by phosphate and active vitamin D substitution. When they are not sufficient or poorly tolerated, patients may be treated by burosumab. Burosumab is an antibody inhibiting FGF23 activity that has been shown to be very efficacious in improving phosphatemia and the clinical symptoms of the disease both in children and adults. In the latter, it decreases joint pain and stiffness and heals fractures. In some cases, due to clinical situations (like pregnancy) or reimbursement reasons, burosumab must be discontinued. There are very few studies on the consequences of burosumab discontinuation. We present the case of a young woman who very strongly improved of her symptoms and healed chronic fractures thanks to burosumab treatment but had to stop it because of discontinuation of reimbursement. Her symptoms relapsed less than 3 months after interrupting it, and she rapidly developed new fractures. Blood tests suggest that FGF23 had accumulated during burosumab treatment, although it could be spurious results due to laboratory technical interferences. The article discusses the literature on this eventual laboratory interferences, and the hypothesis exploring some reasons for FGF23 increase under burosumab treatment.

## Data Availability

The original contributions presented in the study are included in the article/supplementary material. Further inquiries can be directed to the corresponding author.
